# Multidimensional geriatric evaluation in acromegaly: a comparative cross-sectional study

**DOI:** 10.1186/s12877-021-02549-4

**Published:** 2021-10-26

**Authors:** Irene Gagliardi, Sabrina Chiloiro, Maria Vallillo, Marta Bondanelli, Stefano Volpato, Antonella Giampietro, Antonio Bianchi, Laura De Marinis, Maria Chiara Zatelli, Maria Rosaria Ambrosio

**Affiliations:** 1 Section of Endocrinology, Geriatrics & Internal Medicine, Department of Medical Sciences, University of Ferrara, Via Fossato di Mortara 64/B, 44121 Ferrara, Italy; 2 Department of Translational Medicine and Surgery, UOC Endocrinology and Diabetology, Fondazione A Gemelli, IRCCS, Università Cattolica del Sacro Cuore, Largo Agostino Gemelli 8, 00168 Rome, Italy

**Keywords:** Acromegaly, Elderly, Cognition, Disability, Sarcopenia, QoL

## Abstract

**Background:**

Improvement in acromegaly management increased disease survival and prevalence. Evidence regarding acromegaly in older adults are sparse. We aim to explore acromegaly impact on aging process quality.

**Methods:**

Multicenter case-control study conducted on 42 older adults (≥ 65 years) acromegaly patients (ACRO) compared to an age- and gender-matched control group (CTR). Each participant underwent a multidimensional geriatric evaluation.

**Results:**

Mean age in both groups was 73 ± 6 years and female gender was most represented (69%). All comorbidities were more frequent in ACRO than CTR. Thirteen ACRO were in remission and 29 had active disease controlled by medical therapy except for one patient. ACRO showed worse physical performance and mobility skills worsening with age as compared to CTR. ACRO performed poorly in functional status assessment, and age negatively correlated with instrumental and basic daily activities execution. Cognitive evaluation scores were significantly lower in ACRO vs. CTR, worsening with age. No difference was found concerning nutritional and psychological status. Musculoskeletal and bone diseases were more frequent in ACRO than in CTR (52% vs. 12%; 64% vs. 10%; *P* < 0.05) and independently associated with geriatric outcomes in ACRO. ACRO reported a less satisfactory quality of life concerning physical activity and pain, general health, vitality, social activities.

**Conclusions:**

Our study demonstrates increased frailty of older acromegaly patients as compared to non-acromegaly patients with a consequent negative impact on their quality of life. Therefore, it seems advisable to include physical, functional, cognitive, nutritional, and psychological status assessments in routine clinical practice. Further studies are needed to identify the most appropriate geriatric tools.

## Background

Acromegaly is a rare disease caused by growth hormone (GH) hypersecretion mostly due to a pituitary adenoma, with consequent increase in insulin-like growth factor-1 (IGF-1) levels. Acromegaly presents with a progressive and chronic systemic involvement and comorbidities (cardiovascular, respiratory, metabolic, neoplastic, neurological, musculoskeletal) that are responsible for increased morbidity and mortality [1]. Literature reports a higher risk to develop cognitive impairment [2–6], psychopathologies [7–14], poor physical and functional performance [2, 15, 16] with negative effects on global quality of life (QoL). Improvement in diagnostic approaches, surgical techniques, medical therapies over the years has increased acromegaly survival and prevalence in all age groups [17–21]. Recent epidemiological data suggest a reduction in acromegaly mortality with death causes more similar to those of the general population of corresponding age. Therefore, the number of older patients with acromegaly, newly diagnosed or in follow-up, is expected to grow in the coming years. At the same time, the general population’s life span increased [18–24]. We recently reviewed data from the literature to define acromegaly characteristics in the elderly but data regarding the impact of acromegaly disease on the natural aging course are still sparse [17]. Hatipoglu et al. have underlined the importance of a geriatric multidimensional assessment also in older patients with acromegaly since the disease may enhance some aspects of the aging process (cognitive dysfunction, malnutrition risk, reduced physical performance, mood impairment) [2].

In this multicenter study we aim to define how acromegaly can impact on the quality of the aging process as compared to older general population. To this purpose, we performed a multidimensional evaluation of a cohort of Italian older acromegaly patients to assess their clinical, cognitive, functional, nutritional, and psychological status as compared to non-acromegaly older subjects.

## Methods

This multicenter case-control study was conducted in two Italian endocrinological centers (Azienda Ospedaliero Universitaria of Ferrara and Policlinico A. Gemelli of Rome). Older acromegaly patients were recruited among those patients aged ≥65 years old. Acromegaly diagnosis was based on: 1) the presence of typical clinical features, 2) IGF-1 levels higher than age and sex adjusted reference ranges, 3) an abnormal GH response to an oral glucose tolerance test, 4) the presence of a pituitary adenoma. Study population included 42 cases (ACRO). The control group (CTR) included 42 age- and gender-matched subjects randomly selected among patients referred to an endocrinological and internist outpatient clinic (1:1 ratio), with no personal history or clinical suspicion of any pituitary disease. ACRO patients were divided into two groups at the last follow-up: 1) patients in biochemical remission (R-ACRO) presenting normal IGF-1 levels for age and sex without any medical treatment; 2) patients with active disease (A-ACRO), who still needed medical treatments. In the latter group, we further divided controlled A-ACRO patients presenting IGF-1 levels within the age and sex reference range (cA-ACRO) from uncontrolled A-ACRO who displayed still elevated IGF-1 levels despite medical therapy (uA-ACRO).

Demographic data, comorbidities, medications, and anthropometric measures were collected for each patient of both groups. For the ACRO group only, information about IGF-1 levels (expressed as % IGF-1 of the upper limit of the normal range for age, ULN), disease activity at last follow-up, previous and present acromegaly treatment, pituitary adenoma characteristics at diagnosis were gathered from clinical records. Cognitive function was assessed by using the age- and education-adjusted mini-mental state examination (MMSE) test [25]. Functional status was investigated with Lawton’s instrumental activities of daily living (IADL) scale and with Barthel index for basic activities of daily living (BADL), that assess independence in daily living activities [26, 27]. Physical performance, mobility skills, and sarcopenia were tested by using the Timed Up and Go test (TUG), the Short Physical Performance Battery (SPPB) and the Handgrip test to both hands. Cut-offs established by The European Working Group on Sarcopenia in Older People (EWGSOP) were considered to interpret test results and to define sarcopenia presence and severity [28]. Nutritional status was determined by The Mini-Nutritional Assessment Scale-Short Form (MNA-SF), that screens for malnutrition risk [29]. Geriatric depression scale (GDS-15) by Yesavage [30] was used to screen the presence of depressive symptoms, evaluating the degree of depression. The SF-36 [31] test was used to investigate the self-reported measure of health-related quality of patient life, exploring the perception of eight life aspect domains. All tests and their interpretation cut-offs are listed in Table 1. They were administered by two operators (one per center) equally trained in this study protocol.

**Table 1:** Tests employed in the multidimensional evaluation

Dimension assessed	Test	Cut-off and interpretation	References
**Cognitive function**	MMSE	**≥ 24:** normal **18-23:** mild cognitive impairment **11-17:** moderate cognitive impairment **< 10:** severe cognitive impairment	[25]
**Functional status**	IADL	**8 (5 in males):** independence **0:** total dependence	[27]
	BADL	**100:** total independence **91-99:** slight dependence **61-90:** moderate dependence **21-60:** severe dependence **0-20:** total dependence	[26]
**Physical performance** **Mobility skills** ^**a**^**Sarcopenia**	TUG	**< 20 s:** normal	[28]
	SPPB	**> 8:** normal	
	Handgrip test	**≥ 27 (males):** normal **≥ 16 (females):** normal	
**Nutritional status**	MNA-SF	**> 11:** normal **8–11:** risk of malnutrition **≤7:** malnutrition	[29]
**Depression**	GDS-15	**≤ 5:** normal **6-10:** minor depression **> 10:** major depression	[30]
**Quality of life**	SF-36	**100:** best own health perception **0:** worse own health perception	[31]

^a^As defined in the 2018 EWGSOP guidelines [28]

This study is in accordance with the principles set out in the Declaration of Helsinki, has been specifically approved by the Local Ethics Committee (Comitato Etico Indipendente di Area Vasta Emilia Centro, CE-AVEC, at the Policlinico S.Orsola-Malpighi in Bologna) and authorized by the General Director of the Azienda Ospedaliero Universitaria in Ferrara (protocol number CE-AVEC 364/2018/Oss/AOUFe). All subjects read and signed the informed consent form before enrolling in the study.

### Statistical analysis

Categorical variables are presented as absolute values/percentages and Chi-square test was used to compare results. Continuous variables are described by mean values ± standard error of the mean (SE) and were compared by using the Student’s T test in case of a normal distribution or by using the Mann–Whitney U test in case of non-normal distribution. Normality was tested with the Kolmogorov–Smirnov test. Statistical correlation between two continuous variables was evaluated by Pearson’s correlation coefficient. *P* < 0.05 was considered as statistically significant. A multivariate analysis of variance (MANOVA) was performed to evaluate the involvement of comorbidites in the relationship between acromegaly and geriatric outcomes.

## Results

The mean age in both groups was 73 ± 6 years (range 65-91) and female gender was the most represented (69%). Daily medications number was significantly higher in ACRO as compared to CTR (8 ± 3 vs. 3 ± 2), without significant differences regarding antidepressant/antipsychotic drugs. BMI was higher in ACRO, but no significant differences were found concerning weight, waist and hip circumference as compared to CTR (Table 2).

**Table 2:** ACRO and CTR clinical and anthropometric characteristics

Clinical and anthropometric characteristics	ACRO	CTR
**Total (n)**	42	42
**M/F**	13/29	13/29
**Mean age (years ± SE)**	73 ± 0.9	73 ± 0.9
**Medications per day (n ± SE)**	*8 ± 1.2	3 ± 0.5
**Antidepressants / antipsychotics / antiepileptic medications (n, %)**	11 (26%)	5 (12%)
**Weight (Kg ± SE)**	79 ± 2.5	74 ± 1.5
**BMI (Kg/m**^**2**^ **± SE)**	*30 ± 0.9	28 ± 0.6
**∙ < 18.5 (n, %)**	0 (0%)	0 (0%)
**∙ 18.5-24.99 (n, %)**	6 (14%)	10 (23.8%)
**∙ 25 − 29.99 (n, %)**	17 (40%)	22 (52.4%)
**∙ 30-34.99 (n, %)**	10 (24%)	6 (14.3%)
**∙ 35-39.99 (n, %)**	5 (12%)	4 (9,5%)
**∙ > 40 (n, %)**	4 (10%)	0 (0%)
**Waist circumference (cm ± SE)**	137 ± 6.2	132 ± 6
**Hip circumference (cm ± SE)**	141 ± 5.7	139 ± 5.9
**Waist/Hip circumference ratio**	0.97 ± 0.09	0.95 ± 0.09

^*^P < 0.05

ACRO clinical data at diagnosis and at last follow-up are summarized in Table 3.

**Table 3:** ACRO clinical data

**DIAGNOSIS**	
**Mean age (years ± SE)**	58 ± 1.5
**Age ≥ 65 (n, %)**	13 (31%)
**Age ≤ 65 (n, %)**	29 (69%)
**Microadenoma (n, %)**	16 (38%)
**Macroadenoma (n, %)**	26 (62%)
**Extra-sellar extension (n, %)**	12 (29%)
**LAST FOLLOW-UP**	
**Remission (n, %)**	13 (31%)
**Active controlled disease (n, %)**	28 (67%)
**Active uncontrolled disease (n, %)**	1 (2%)
**Mean % IGF-1 ULN ± SE**	-29 ± 4.5%
**Mean GH levels (ng/ml ± SE)**^**a**^	0.98 ± 0.1
**Ongoing acromegaly therapy**	
**∙ None (n, %)**	13 (31%)
**∙ SSA (n, %)**	15 (35.7%)
**∙ PEG (n, %)**	2 (4.7%)
**∙ DA (n, %)**	1 (2.4%)
**∙ PEG + DA (n, %)**	1 (2.4%)
**∙ SSA + DA (n, %)**	4 (9.5%)
**∙ SSA + PEG (n, %)**	5 (11.9%)
**∙ SSA + PEG + DA (n, %)**	1 (2.4%)
**Previous acromegaly treatment**	
**∙ Surgery (n, %)**	29 (69%)
**∙ RT (n, %)**	1 (2%)
**∙ GK (n, %)**	2 (5%)
**∙ SSA (n, %)**	14 (33%)
**∙ PEG (n, %)**	4 (10%)
**∙ DA (n, %)**	2 (5%)

*RT* conventional radiotherapy, *GK* gamma knife radiotherapy, *SSA* somatostatin analogue, *PEG* pegvisomant, *DA* dopamine agonists

^a^Available in 33 out of 42 patients (9 patients were under Pegvisomant treatment)

**Fig. 1:**
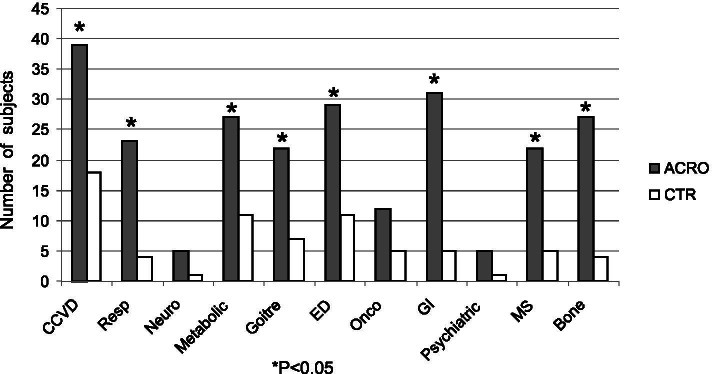
ACRO and CTR comorbidities. CCVD = cardio/cerebro-vascular disease (arrhythmia, hypertension, valvulopaties, ischemic heart disease, cerebral aneurysm, stroke); Resp = respiratory (OSAS); Neuro = neurological; Metabolic = metabolic impairments (diabetes mellitus, dyslipidemia); ED = endocrine dysfunctions (hypothyroidism, hypogonadism, hypoadrenalism, hyperprolactinemia); Onco = oncological; GI = digestive system diseases (intestinal polyposis, gastritis, hepatic steatosis, cholelithiasis, diverticulosis); MS = musculoskeletal diseases; Bone = bone diseases (osteopenia/osteoporosis). *P < 0.05 ACRO vs. CTR

ACRO, 13 were in remission at the last evaluation (R-ACRO) and 29 presented active disease (A-ACRO), all controlled by medical therapy except for one patient with uncontrolled active disease. Concerning pituitary function, hypoadrenalism, hypothyroidism, hypogonadism and hyperprolactinemia were present in 21, 50, 17 and 10% of ACRO patients, respectively. Hyperprolactinemia was treated with dopamine agonists (DA). Replacement therapy was provided to all patients except for 3 hypogonadal male patients due to comorbidities. Cardio- and cerebro-vascular diseases, respiratory diseases, metabolic impairments, goiter, endocrine dysfunctions, gastrointestinal diseases, musculoskeletal complications, and bone diseases were significantly more frequent in ACRO as compared to CTR (Fig. 1). Regarding endocrine disfunctions, CTR presented only primary hypothyroidism which was well replaced on levothyroxine therapy.

Cognitive evaluation showed significantly lower MMSE scores in ACRO as compared to CTR (Table 4); both groups had mean MMSE scores in the normal range. Indeed, according to MMSE test cut-off, no differences were detected concerning the prevalence of cognitive impairment among the two groups. Scores negatively correlated with age in ACRO (*r* = − 0.25, *P* < 0.01) and positively in CTR (*r* = 0.37, *P* < 0.01).

**Table 4:** ACRO and CTR multidimensional evaluation

Multidimensional evaluation	ACRO	CTR
**MMSE**		
Score ± SE	*26 ± 0.8	28 ± 0.3
Normal (n, %)	35 (83.3%)	41 (98%)
Mild cognitive impairment (n, %)	5 (11.9%)	1 (2%)
Moderate cognitive impairment (n, %)	1 (2.4%)	0 (0%)
Severe cognitive impairment (n, %)	1 (2.4%)	0 (0%)
**IADL**		
Score ± SE	*5 ± 0.3	7 ± 0.2
Total independence (n, %)	20 (48%)	29 (69%)
Total dependence (n, %)	0 (0%)	0 (0%)
**BADL**		
Score ± SE	*88 ± 3.5	99 ± 0.3
Total independence (n, %)	27 (64.3%)	37 (88.1%)
Slight dependence (n, %)	4 (9.5%)	2 (4.8%)
Moderate dependence (n, %)	7 (16.6%)	3 (7.1%)
Severe dependence (n, %)	2 (4.7%)	0 (0%)
Total dependence (n, %)	2 (4.7%)	0 (0%)
**TUG**		
Seconds ± SE	*16 ± 1.1	11 ± 0.8
Sarcopenia (n, %)	*14 (36%)	2 (5%)
**SPPB**		
Score ± SE	*7 ± 0.5	10 ± 0.3
Sarcopenia (n, %)	*28 (70%)	6 (15%)
**HANDGRIP TEST**		
Dominant hand (Kg ± SE)	22 ± 1.6	24 ± 3.9
Non dominant hand (Kg ± SE)	17 ± 1.6	20 ± 1.2
Sarcopenia (n, %)	14 (36%)	9 (21%)
**MNA-SF**		
Score ± SE	11 ± 0.3	12 ± 0.3
Normal (n, %)	22 (52%)	29 (69%)
Malnutrition risk (n, %)	18 (43%)	13 (31%)
Malnutrition (n, %)	2 (5%)	0 (0%)
**GDS-15**		
Score ± SE	5 ± 0.5	4 ± 0.5
Normal (n, %)	24 (57%)	30 (71%)
Minor depression (n, %)	16 (38%)	12 (29%)
Major depression (n, %)	2 (5%)	0 (0%)

^*^P < 0.05 ACRO vs. CTR

Functional assessment demonstrated significantly lower IADL and BADL scores in ACRO as compared to CTR, without differences in functional dependence levels. In ACRO both scores negatively correlated with age (*r* = − 0.29, *P* < 0.01; *r* = − 0.41, *P* < 0.01). On the contrary, in CTR age positively correlated with IADL (*r* = 0.07, *P* < 0.01) and negatively with BADL (*r* = − 0.29, *P* < 0.01).

Regarding physical performance status and sarcopenia, ACRO obtained poorer results at TUG and SPPB tests as compared to CTR (Table 4). No difference was observed at the handgrip test and the strength developed by both hands did not differ between the two groups. In ACRO age negatively correlated with both SPPB test (*r* = − 0.37, *P* < 0.01) and handgrip test (dominant hand *r* = − 0.29 and non-dominant hand *r* = − 0.21, *P* < 0.01). In addition, age positively correlated with time to complete the TUG test (*r* = 0.17, *P* < 0.01). In CTR only a negative correlation with the dominant handgrip test was found (*r* = − 0.18, *P* < 0.01). Nutritional status did not differ between groups on the basis of MNA-SF test scores. Scores become worse with age in ACRO but not in CTR (*r* = − 0.30 vs. 0.06, *P* < 0.01). Among ACRO, mean MNA-SF scores were significantly lower in R-ACRO than A-ACRO (*P* < 0.05), with a higher number of patients at risk of malnutrition as compared to A-ACRO (69%, *P* < 0.05). Depression screening did not reveal any significant differences between the groups. GDS-15 scores did not show any correlation with age in both groups. All evaluations are summarized in Table 4.

**Fig. 2:**
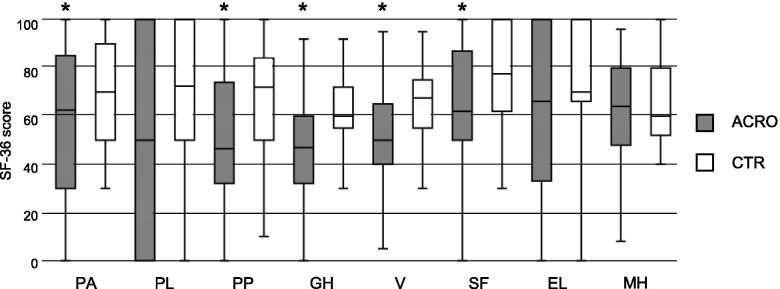
ACRO and CTR SF-36 questionnaire. PA = physical activity; PL = physical limitations; PP = physical pain; GH = general health; V = vitality; SF = social functioning; EL = emotional limitations; MH = mental health. * *P* < 0.05

ACRO presented less satisfactory scores in 5 out of 8 SF-36 questionnaire domains as compared to CTR: physical activity, physical pain, general health, vitality, social activities (Fig. 2). Correlation with age was negative in all 8 fields in ACRO (*P* < 0.05). On the contrary, in CTR this correlation was significantly positive in the general health, vitality, and mental health fields. The multivariate analysis showed the significant association of specific comorbidities with geriatric outcome scores in ACRO (Table 5). No differences were observed comparing R-ACRO and A-ACRO in all items, except for MNA-SF scores.

**Table 5:** Comorbidities associated to geriatric outcome scores in ACRO by multivariate analysis

	IADL	BADL	TUG	SPPB
Cardio and cerebrovascular disease OR (IC 95%)	1.89* (1.17 - 3.03)	1.23 (0.77 - 1.98)	0.83 (0.51 - 1.33)	0.99 (0.62 - 1.59)
Respiratory disease OR (IC 95%)	0.39* (0.24 - 0.62)	0.60* (0.37 - 0.96)	1.91* (1.19 - 3.06)	0.54* (0.34 - 0.88)
Metabolic impairment OR (IC 95%)	0.95 (0.59 -1.53)	1.08 (0.67 - 1.74)	0.60 (0.38 – 0.97)	1.40 (0.87 - 2.25)
Goitre OR (IC 95%)	0.87 (0.54 - 1.40)	0.78 (0.49 - 1.26)	0.90 (0.56 - 1.44)	0.74 (0.46 - 1.19)
Endocrine dysfunctions OR (IC 95%)	1.19 (0.74 - 1.91)	0.34* (0.21 - 0.54)	0.63* (0.39 - 1.01)	1.05 (0.65 - 1.69)
Oncological disease OR (IC 95%)	1.43 (0.89 - 2.30)	1.23 (0.77 - 1.98)	0.85 (0.53 - 1.36)	1.43 (0.89 - 2.30)
Digestive diseases OR (IC 95%)	0.86* (0.54 - 1.39)	0.85* (0.53 - 1.37)	1.52* (0.95 - 2.45)	0.69* (0.43 - 1.10)
Psychiatric OR (IC 95%)	0.49 (0.31 - 0.79)	0.48 (0.30 – 0.77)	1.08 (0.67 - 1.74)	0.84 (0.52 - 1.34)
Musculoskeletal diseases OR (IC 95%)	0.89 (0.55 - 1.42)	0.89 (0.56 - 1.44)	1.73* (1.08 - 2.78)	0.89 (0.55 - 1.42)
Bone diseases OR (IC 95%)	1.03* (0.64 - 1.65)	1.36* (0.85 - 2.19)	1.11* (0.69 - 1.79)	0.67* (0.42 – 1.08)

^*^P < 0.05

## Discussion

Our study demonstrates an increased frailty of older acromegaly patients as compared to non-acromegaly older patients, especially concerning cognitive function, functional status, and physical performance, with consequent negative impact on their quality of life.

In our study the ACRO group presented an average age of 58 ± 10 years at diagnosis. A third of them had been diagnosed after 65 years of age, possibly supporting the hypothesis that acromegaly has a mild clinical phenotype in the elderly patient [17]. Two thirds were diagnosed before 65 years of age, therefore we cannot exclude an influence of disease duration on our results. Indeed, it is well consolidated that disease duration influences comorbidities development and worsening [1]. ACRO group was predominantly represented by females, in agreement with recent Italian series [18, 22, 23, 32]. Pituitary adenomas were mostly macroadenomas, in contrast with literature data reporting that older patients generally carry smaller tumors as compared to younger patients [17]. This difference was further underlined by the finding that macroadenomas were more represented than microadenomas when considering only patients diagnosed with acromegaly after 65 years old. Therefore, our data indicate that age at diagnosis is not always predictive of tumor size. However, all macroadenomas were enclosed in the sellar space, in keeping with previous series [17] suggesting a milder behaviour of these tumors.

Our study shows a significant difference in BMI between ACRO and CTR, but waist/hip circumference ratio does not differ in the two groups, suggesting a similar fat tissue distribution. However, the available data do not allow to confirm this hypothesis and further studies are needed.

Most patients presented an active disease but controlled by medical treatment, mostly somatostatin analogs (SSA), in line with the guidelines [33]. Therefore, our ACRO group was homogeneous in terms of biochemical disease control, allowing us to hypothesize that the worse performance of ACRO vs. CTR was not due to aging nor to poor disease control, but possibly to the consequences of GH/IGF-1 excess before treatment. These data are in line with previous reports, showing that cognitive and psychological performance does not improve in acromegaly patients over time despite disease control [4, 9, 34, 35].

ACRO presented a more complex clinical picture than CTR, as indicated by the significantly higher number of medications per day and the more frequent comorbidities. Both acromegaly and aging are associated to comorbidities development, but our data suggest that acromegaly disease might be an additional factor negatively influencing older adults health. We observed a more important involvement of the musculoskeletal system in ACRO than in CTR. Indeed, arthropathy and osteoporosis are often described since the diagnosis of acromegaly and they usually persist also after remission [16, 36–38]. Such evidence might partially explain our results derived from physical and functional evaluations. Indeed, ACRO obtained worse scores in the TUG and SPPB tests, describing worse physical performance and mobility skills. On the contrary, handgrip test results were similar in ACRO and CTR, in keeping with Füchtbauer et al. who did not find proximal muscle weakness in patients normalized after acromegaly remission. We also found that bone and musculoskeletal diseases are independently associated with geriatric outcome scores in ACRO. Our results are in agreement with the study by da Silva Homem et al., who found a greater risk of falls and a worse performance concerning balance control, and peripheral muscle function in 17 acromegalic older adults (age range: 63-73 years) compared with 20 paired controls [15]. Furthermore, we demonstrated that scores related to physical performance, mobility skills and sarcopenia worsen with age, suggesting a synergistic effect between aging and acromegaly, accounting for an increased frailty in older acromegaly patients. This hypothesis is further strengthened by the evidence that 3 out of 42 ACRO patients had been unable to complete the tests evaluating physical performance. Contrary to our findings, Hatipoglu et al. found no difference between the ACRO and CTR aging groups, by performing 2 out of 3 tests among those we employed. These differences may be due to the different number of evaluated patients; in addition, comorbidities were not fully characterized [2]. Furthermore, evidence regarding the relationship between acromegaly, sarcopenia and musculoskeletal status is still conflicting and very few data are available in the elderly, impairing the evaluation of the role of quantitative and qualitative muscle dysfunction [17].

Our study shows that functional status assessed by IADL and BADL is worse in ACRO vs. CTR, similarly to what found by Hatipoglu et al. [2]. Furthermore, in ACRO aging was negatively correlated with performance in instrumental and basic daily activities, possibly due to the worse musculoskeletal system condition and the presence of other comorbidities, as shown by the multivariate analysis. Our data support the hypothesis that musculoskeletal impairment is the primary source of physical pain and disability with negative impact on daily social activities. On the other hand, the employed evaluation was unable to discriminate the degree of dependence, in keeping with Fatti et al. [16] and suggesting that other tools are necessary to better identify those patients who need more assistance.

Cognitive dysfunction in acromegaly ranges from 2 to 33% of patients and is mostly represented by memory and attention impairments. Neurocognitive alterations persist after surgical remission [4] suggesting that hormonal disease control is not sufficient to restore cognitive functions [34]. In keeping with these data, our experience shows that also in ACRO older patients cognitive performance is significantly impaired, despite disease control, worsening with age. Similarly to what found for functional status, MMSE was unable to capture the degree of cognitive impairment, probably due to the reduced size of our sample. Our data suggest that investigating cognitive function is appropriate, especially in older patients, considering MMSE scores as a preliminary evaluation.

Regarding nutritional and psychological evaluation, ACRO and CTR were similar in our settings. Hatipoglu et al., instead, described a worse nutritional status with a greater risk of malnutrition in acromegaly compared to the general older population, independently of GH/IGF-1 levels or disease activity [2]. These results suggest that MNA-SF and GDS-15 tests may not be sensitive enough to detect nutritional and psychological differences in controlled acromegaly versus normal aging subject. Further studies with larger cohorts and more sensitive tools are desirable.

Previous studies have widely documented the association between acromegaly and a poor QoL [6, 35, 39]. We found that QoL is worse in 5 out of 8 domains of the SF-36 questionnaire, confirming that acromegaly negatively impacts QoL also in the aging patient. AcroQoL, a disease-specific QoL questionnaire [40], is not applicable in our settings since it cannot measure QoL in CTR.

Finally, no relevant differences were found between A-ACRO and R-ACRO, but definitive conclusions cannot be derived due to the restricted number of the subsamples.

The multicenter design of our study allowed us to collect a consistent number of older acromegaly patients, considering that acromegaly is a rare disease. However, the main limitation of our study still remains the small samples size which might reduce the statistical power of our analysis. Two operators (one per center) equally trained in this study protocol have tested patients and controls in the two centers, making results more homogeneous. The multidimensional geriatric assessment of our study consists of simple tools readily available in routine clinical practice and extensively used in the geriatric general population. However, even if we derived significant conclusions from their applications, all tests did not perform equally in the acromegaly population. From our experience, the TUG and SPPB tests should be considered as useful tools to evaluate performance and sarcopenia status in acromegalic patients during routine clinical practice. They are easy to carry out and they are time and cost saving. However, body composition should also be explored besides patient performance, with specific tools (e.g. BIA, DEXA, MRI or CT).

## Conclusions

Our multidimensional evaluation suggests that older acromegaly patients are much frailer than the general geriatric population, with a negative impact on their daily quality of life, supporting the indication to submit acromegaly patients to geriatric evaluation at an earlier age as compared to the general population. Our results suggest that disease management should take care also of such typical geriatric issues that could appear earlier and more importantly in acromegaly. Older acromegaly patients may indeed need assistance and support in their daily activities more often.

Therefore, it seems advisable to include physical, functional, cognitive, nutritional and psychological status assessments in routine clinical practice. However, other studies are needed to identify the most appropriate and efficient geriatric tools for acromegaly disease evaluation.

## Data Availability

The datasets generated and analyzed during the current study are available from the corresponding author on reasonable request.

## Abbreviations

GH: Growth hormone
IGF-1: Insulin-like growth factor-1
ACRO: Patient group
CTR: Control group
MMSE: Mini-mental state examination test
IADL: Lawton’s instrumental activities of daily living scale
BADL: Barthel index for basic activities of daily living
TUG: Timed Up and Go test
SPPB: Short Physical Performance Battery test
MNA-SF: Mini-Nutritional Assessment Scale-Short Form
GDS-15: Geriatric depression scale
